# Green Ultrasound Assisted Extraction of *trans* Rosmarinic Acid from *Plectranthus scutellarioides* (L.) R.Br. Leaves

**DOI:** 10.3390/plants8030050

**Published:** 2019-02-27

**Authors:** Duangjai Tungmunnithum, Laurine Garros, Samantha Drouet, Sullivan Renouard, Eric Lainé, Christophe Hano

**Affiliations:** 1 Laboratoire de Biologie des Ligneux et des Grandes Cultures, INRA USC1328, Orleans University, 45067 Orléans Cedex 2, France; laurine.garros@etu.univ-orleans.fr (L.G.); samantha.drouet@univ-orleans.fr (S.D.); eric.laine@univ-orleans.fr (E.L.); 2 Bioactifs et Cosmetiques, CNRS GDR 3711 Orleans, 45067 Orléans Cedex 2, France; 3 Department of Pharmaceutical Botany, Faculty of Pharmacy, Mahidol University, Bangkok 10400, Thailand; 4 Institut de Chimie Organique et Analytique, CNRS UMR731, Orleans University, 45067 Orléans Cedex 2, France; 5 Institut de Chimie et de Biologie des Membranes et des Nano-objets, CNRS UMR 5248, Bordeaux University, 33600 Pessac, France; sullivan.renouard@u-bordeaux.fr

**Keywords:** *Plectranthus scutellarioides*, *trans*-rosmarinic acid, *Lamiaceae*, green extraction, ultrasound, antioxidant, antimicrobial

## Abstract

Painted nettle (*Plectranthus scutellarioides* (L.) R.Br.) is an ornamental plant belonging to *Lamiaceae* family, native of Asia. Its leaves constitute one of the richest sources of *trans*-rosmarinic acid, a well-known antioxidant and antimicrobial phenolic compound. These biological activities attract interest from the cosmetic industry and the demand for the development of green sustainable extraction processes. Here, we report on the optimization and validation of an ultrasound-assisted extraction (USAE) method using ethanol as solvent. Following preliminary single factor experiments, the identified limiting extraction parameters (i.e., ultrasound frequency, extraction duration, and ethanol concentration) were further optimized using a full factorial design approach. The method was then validated following the recommendations of the association of analytical communities (AOAC) to ensure the precision and accuracy of the method used to quantify *trans*-rosmarinic acid. Highest *trans*-rosmarinic acid content was obtained using pure ethanol as extraction solvent following a 45-minute extraction in an ultrasound bath operating at an ultrasound frequency of 30 kHz. The antioxidant (in vitro radical scavenging activity) and antimicrobial (directed toward *Staphylococcus aureus* ACTT6538) activities were significantly correlated with the *trans*-rosmarinic acid concentration of the extract evidencing that these key biological activities were retained following the extraction using this validated method. Under these conditions, 110.8 mg/g DW of *trans*-rosmarinic acid were extracted from lyophilized *P. scutellarioides* leaves as starting material evidencing the great potential of this renewable material for cosmetic applications. Comparison to other classical extraction methods evidenced a clear benefit of the present USAE method both in terms of yield and extraction duration.

## 1. Introduction

**Figure 1:**
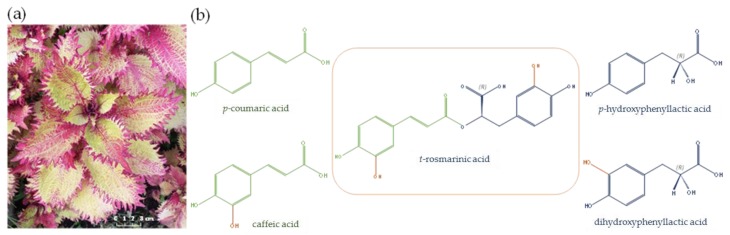
(**a**) Painted nettle (*Plectranthus scutellarioides* (L.) R.Br.) leaves morphology; (**b**) Chemical structures of *p*-coumaric acid, caffeic acid, *p*-hydrophenyllactic acid, dihydrophenyllactic acid, and *trans*-rosmarinic acid (picture taken by C.H.).

*Plectranthus scutellarioides* (L.) R.Br. or painted nettle (Figure 1a) is an ornamental and medicinal plant, mainly found in Asia, currently spread over America, Southern Africa, and Europe [1,2]. Its synonym, *Coleus blumei* Benth. is also well-known. This plant is a species member of the *Lamiaceae* family, widely cultivated as an ornamental plant due to the beauty of its leaves. Furthermore, local people in many countries have long been used *P. scutellarioides* as an herbal medicine. For example, local Indian people in Arunachal Pradesh use hot water extract of *P. scutellarioides* mixing with fruit juice to apply on the skin in order to decrease undesirable symptoms after scorpion bite [3]. In addition, Tlanchinol people in Mexico use the infusion of this plant for gastrointestinal diseases treatment [4]. Interestingly, its leaves constitute one of the richest sources of an antioxidant and antimicrobial phenolic compound, namely *trans*-rosmarinic acid (RA) (Figure 1b) [5,6,7,8].

From a strict chemical point of view, RA is a caffeic acid and dihydrophenyllactic acid ester; however, biologically, its biosynthetic precursors are *p*-coumaric acid and *p*-hydroxyphenyllactic acid, respectively (Figure 1b). RA has been identified as one of the main bioactive compounds in many *Lamiaceae* from the Nepetoideae subfamily [8], and beside *P. scutellarioides* other *Plectranthus* species have been described for their accumulation of RA such as *P. barbatus* [9,10,11,12], *P. vericallatus* [9,12], *P. madagascarensis* [12,13], *P. ecklonii* [9], *P. fructicosus* [9], *P. lanuginosus* [9], *P. hadiensis* [12], *P. neochilus* [12], *P. amboinicus* [14], or *P. ornatus* [15]. These studies focusing on the biological activities associated with the presence of RA in the extracts obtained from these *Plectranthus* species or comparison of RA content variation among some of these species. Indeed, a number of relevant biological activities have been ascribed to this natural compound among which antioxidant [16], antimicrobial [7], anti-inflammatory [17], antimutagenic [8], antiagiogenic [8], neuroprotective [18], Alzheimer disease preventive action [19] with acetylcholinesterase inhibition capacity [10,11].

Antioxidant action is of particular interest since excessive accumulation of free radicals could constitute a starting point or aggravating factor for many diseases though their potential damages on membrane lipids, DNA, and proteins. Today, natural antioxidants are considered as potential safer and efficient drugs to prevent a wide range of diseases resulting from oxidative stress [20]. Indeed, damaging effects on health, including carcinogenesis, of their synthetic antioxidant and preservative counterparts have been pointed out [21]. As a consequence, the uses of some of these synthetic compounds are now strictly regulated, some have been removed from the ‘generally recognized as safe’ (GRAS) list and are now forbidden for food applications in Japan, Canada, and Europe [22]. Natural compounds have therefore attracted attention because of their potential for application to the food, cosmetic and pharmaceutical industries as natural preservatives because of their antioxidant and antibacterial activities [23,24]. However, the development of effective extraction methods of these natural compounds is necessary. Many extraction methods have been developed to extract natural antioxidants from various naturally occurring matrices based on maceration extraction, Soxhlet extraction, microwave assisted extraction, or ultrasound-assisted extraction (USAE) [25,26,27]. Green extraction technologies have attracted high interest in modern industries over the last decade and ultrasound-assisted extraction (USAE) is now considered as one of the most efficient energy-saving process in terms of duration, selectivity, and reproducibility, operating under soft- to mid-extraction conditions [25]. The improvement of extraction efficiency obtained using USAE is reported to rely on both acoustic cavitation and mechanical effects [25]. Indeed, ultrasounds (US) produce an acoustic cavitation effect facilitating the penetration of the extraction solvent. Consequently, an easier release of the intracellular content of the plant material is observed thank to a greater solvent agitation resulting in an increased surface contact between the solvent and the target compound as well as an enhanced solubility of the target compound into the extraction solvent [25].

To date, most of the studies dealing with RA production from *P. scutellarioides* have focused on the elucidation of its biosynthetic pathway [8] and/or biotechnological approaches to improve its production in planta using in vitro technologies [5,7,8,28,29,30]. Little attention has been paid to the optimization of its extraction from the leaves of this ornamental plant. This ornamental plant is known as easy to propagate by cuttings and high renewable biomass production of leaves can be obtained through basic horticultural approaches, therefore the development of green extraction of RA using this raw starting material for potential cosmetic applications could be very promising. In the literature, some studies have reported on the optimization of USAE of RA from the leaves of other *Lamiaceae* plants such as *Hyssopus cuspidatus* [31], *Rosmarinus officinalis* [32,33], *Melissa officinalis* [34], *Mentha piperita*, *Mentha longifolia*, or *Ocimum basilicum* [35]. RA contents reported by these studies evidenced a strong matrix effect. Plant matrix can have considerable effects (so-called matrix effects) both on the optimal extraction conditions and the resulting extraction yields [36]. This matrix effect depends on the plant species, plant origins, organs or tissue used, drying and storage conditions, and so on. For example, RA obtained following USAE of *Rosmarinus officinalis* dried leaves greatly varied according to the extraction conditions used, as determined by three independent studies [32,33,37], thus evidencing the necessity to specifically optimize and validate the extraction conditions for each plant matrix. In the present study, our goals were to develop and validate a green USAE protocol of RA from *P. scutellarioides* leaves for future cosmetic applications as natural antioxidant and natural preservative using ethanol as green extraction solvent. For this purpose, in order to extract high amount of RA from *P. scutellarioides* leaves we have determined of the most critical extraction parameters—among ethanol concentration, US frequencies, solvent to material ratio, extraction temperature and extraction duration, applied a factorial design of experiment taking into account the possible interaction between the limiting extraction parameters, determined the optimal extraction conditions after statistical analysis and using 3D surface plots, and validated the optimal extraction conditions. Then antioxidant and antibacterial activities of each extract were determined using, the DPPH assay and a validated 96-well plate assay for the monitoring of *Staphylococcus aureus* ACTT6538 growth inhibition, respectively. The efficiency of this optimized and validated extraction protocol was finally compared to the conventional heat reflux extraction procedures.

## 2. Results and Discussion

### 2.1. Preliminary Single Factor Experiments

The relative effect of different independent parameters on the extraction yield of RA from *P. scutellarioides* leaves was first evaluated through single-factor experiments. Here, the effects of several parameters described as important in the literature have been studied: ethanol concentration in aqueous solution, extraction time, extraction temperature, ultrasound frequency, and solvent-to-material ratio (S/M ratio). The main objective of these preliminary single factor experiments was to determine the main limiting factors that will be then applied in the design of experiment in order to evaluate the interaction effect of these extraction parameters.

**Figure 2:**
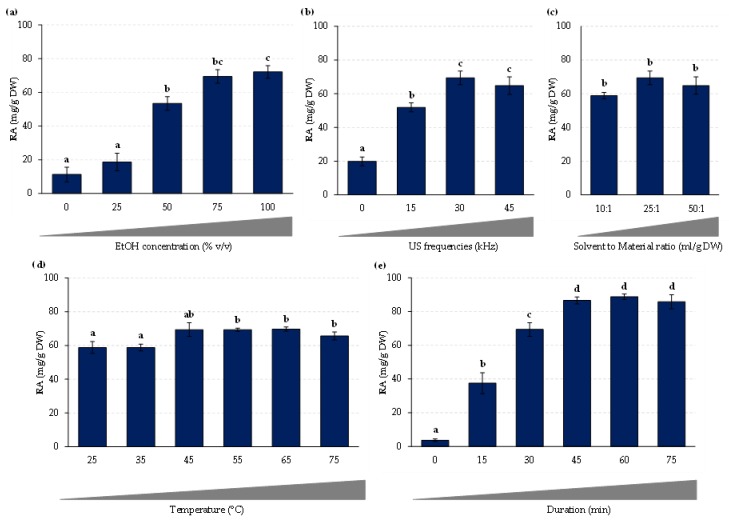
RA contents extracted from *P. scutellarioides* leaves as of function of (**a**) ethanol concentration, (**b**) ultrasound frequency, (**c**) solvent to material ratio, (**d**) extraction temperature, and (**e**) extraction duration. For a complete description of the extraction conditions, see text. Values are means ± SD of 3 independent replicates. Different letters (a–d) represent significant differences between the various extraction conditions (*p* < 0.05).

As part of a green chemistry approach development, the choice of extraction solvent is one of the crucial parameters to take into account. Various organic solvents have commonly been used to extract antioxidant polyphenols from a various plant matrix, such as methanol, ethanol, and acetone [26]. Among these solvents, ethanol is non-toxic to humans and environmentally friendly. Its extraction efficiency can further be improved by mixing it with water, thus making it able to extract a wide range of phenolic compounds. These two universal solvents also present the great advantage of being inexpensive and are therefore widely used in the agri-food and/or cosmetic industries [25,26,27]. For all these reasons, we chose ethanol as the extraction solvent and tested the influence of ethanol concentration in aqueous solution on the extraction of RA. Preliminary experiments were conducted with various concentrations of aqueous ethanol solutions (0, 25, 50, 75, and 100% (*v*/*v*)) using fixed S/M ratio (25:1 mL/g DW), extraction time (30 min), sonication frequency (30 kHz), and extraction temperature (45°C). The results shown in Figure 2a indicate that the RA extraction yield increases with the ethanol concentration reaching a peak (82.1 ± 6.5 mg / g DW) with pure ethanol (100%). The reasons for this could be related to the solubility of RA, which LogP of 2.4 indicates higher solubility in octanol than in water, and to the polarity of ethanol.

It is well established that the ultrasound frequency could greatly influence the extraction efficiency through cavitation effect and by influencing diffusion coefficient of the targeted compounds, resulting in the solubility improvement of the target compound in the extraction solvent. From this observation, it appears that the extraction efficiency could be improved by an increase of the ultrasonic frequency. Nevertheless, high ultrasound frequency can, on the contrary, lead in some conditions to the degradation of the bioactive compounds and consequently considerably reduce the extraction yield [25,26,27]. Therefore, the ultrasound frequency must be precisely optimized. The effect of different ultrasound frequency (0, 15, 30, and 45 kHz) on the extraction yield of RA was thus next evaluated using fixed parameters set as follows: ethanol concentration 100%, S/M ratio 25:1 mL/g DW, extraction time 45 min, and extraction temperature of 45 °C. According to the results presented in Figure 2b, application of ultrasound frequency of 30 and 45 kHz here improved the RA extraction.

Different solvent to material (S/M) ratios were then evaluated (10:1, 25:1, and 50:1 ml of pure ethanol per gram of DW material). These experiments were conducted using fixed ethanol concentration (100% (*v*/*v*)), extraction time (30 min), sonication frequency (30 kHz), and extraction temperature (45 °C). The results shown in Figure 2c indicate that the RA extraction yield was not significantly influenced by the S/M ratio.

The effect of different extraction temperature (25, 35, 45, 55, 65, and 75°C) on the extraction yield of RA was thus next evaluated using fixed parameters set as follows: ethanol concentration 100%, S/M ratio 25:1 mL/g DW, extraction time 45 min and ultrasound frequency 45 kHz. According to the results presented in Figure 2d, the extraction efficiency is here only slightly influenced by the temperature parameter. Indeed, only a slight increase in the extraction yield was noted by increasing the extraction temperature from 25 to 65°C, whereas increasing extraction temperature to 75 °C using these conditions resulted in a decreased RA yield. It is accepted that an excessive temperature coupled to ultrasound can cause the degradation of the target compound [25,26,27]. From these results, extraction temperature did not appear as a limiting parameter for the extraction of RA from *P. scutellarioides* leaves.

The effect of extraction duration on the RA extraction efficiency was studied for a duration ranging from 0 to 60 min, with other parameters fixed at 100% for ethanol concentration, 25:1 mL/g (DW) for S/M ratio, 45°C as the extraction temperature and 30 kHz ultrasound frequency. The results depicted in Figure 2e indicate that the RA extraction yield increases with the extraction time in a first phase reaching a plateau after 45 min. We noted that the observed increase between 45 and 60 min was not statistically significant. Under these conditions, the maximum extraction efficiency could therefore be obtained after 45 min. It has been described that prolonged ultrasound can lead to the degradation of antioxidant phenolic compounds [25,26,27]. Consequently, extraction time was set to 45 min for the following experiments.

### 2.2. Develoment of a Multifactorial Approach

Experimental factorial design associated with statistical analysis and 3D surface response plots have proven their efficiencies in the precise and rapid optimization of extraction protocols by taking into account the possible interaction between independent variables which is not the case when developing an extraction protocol using a single factor approach [38]. Here, from our preliminary experiments, three influencing variables were selected to optimize RA extraction from *P. scutellarioides* leaves using a factorial design approach: aqueous ethanol concentration (X_1_, ranging from 50 to 100 % (*v*/*v*)), US frequency (X_2_, ranging from 15 to 45 kHz) and extraction duration (X_3_, ranging from 15 to 45 min). The coded levels and actual experimental values of each independent variables are presented in Table 1. Note that, according to our preliminary experiments, a solvent to material ratio of 25:1 ml/g DW and an operating temperature of 45°C were chosen.

Here, a full factorial design was chosen for the optimization because of the high reproducibility of the obtained results due to the real measurement of a extensive number of experimental conditions compared to other design of experiments approaches [39]. For the experiment, the 27 different conditions (run ID) using independent process variables were randomized (run order), tested as independent triplicates, and the resulting extracted RA content evaluated by HPLC (Table 2).

Under these extraction conditions, we found RA contents extracted from freeze-dried *P. scutellarioides* leaves ranging from 2.4 (Obs22) to 110.8 (Obs24) mg/g DW. Following multiple regression analysis, the RA content (Y) as a function of the different variables (X_1_, X_2_, and X_3_) was represented by the following second order polynomial equation: Y = 73.22 + 31.74X_1_ + 6.70X_2_ + 2.15X_3_ − 17.60X_1_^2^ − 8.68X_2_^2^ − 0.65X_3_^2^ + 6.34X_1_X_2_ + 2.11X_1_X_3_ − 4.11X_2_X_3_ (Table 3).

As a result of the statistical analysis, the linear coefficients X_1_ and X_2_, the quadratic coefficients X_1_^2^ and X_2_^2^ as well as the interaction coefficient X_1_X_2_ appeared to significantly influence the extraction efficiency of RA from freeze-dried *P. scutellarioides* leaves. On the contrary, the other linear (X_3_), quadratic (X_3_^2^) and interaction (X_1_X_3_ and X_2_X_3_) coefficients were insignificant (*p* > 0.05). Therefore, ethanol concentration (X1) as well ultrasound frequency (X2) and their interaction appeared to influence greatly the extraction efficiency over extraction duration.

Analysis of variance (ANOVA) result and the fit of the obtained model are listed in Table 4. The model was highly significant as indicated by the high *F*-value (22.72) and the low *p*-value (*p* < 0.0001), but also by the low lack of fit *F*-value (0.87) and its non-significant associated *p*-value (*p* > 0.05). The RA contents predicted vs. the experimentally measured RA contents are plotted in Figure S1 and confirmed the high precision of the model. The determination coefficient R^2^ of 0.924 and the adjusted R^2^ of 0.883 both confirmed this model is adequate to predict the RA extraction yield from freeze-dried *P. scutellarioides* leaves. In addition, the variation coefficient value (CV = 0.79%) also indicated the adequacy between the model and experimental values.

**Figure 3:**
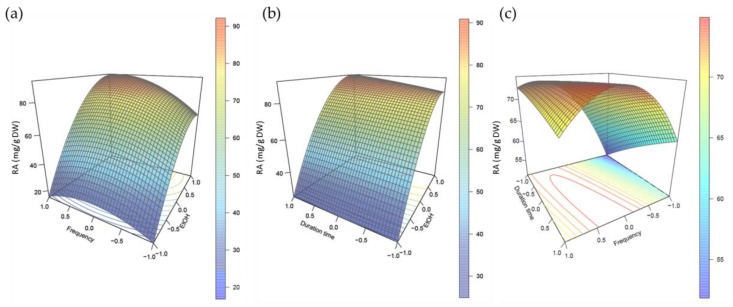
Predicted surface response plots of the RA extraction yield as a function of (**a**) ultrasound frequency and ethanol concentration, (**b**) extraction duration and ethanol concentration, and (**c**) extraction duration and ultrasound frequency.

Here, the 3D plots accounted for the complexity of the USAE of RA from lyophilized *P. scutellarioides* leaves. All the linear coefficients (ethanol concentration, ultrasound frequency, and extraction duration) as well as the interaction coefficients X_1_X_2_ (ethanol concentration and ultrasound frequency) and X_1_X_3_ (ethanol concentration and extraction duration) of the second-order polynomial equation obtained were positive, indicating that increasing these parameters exerted a global favorable action on RA extraction. In good agreement with this observation, the resulting 3D plots confirmed the favorable positive effects on RA extraction resulting in the increase of ethanol concentration combined with the increased extraction duration or ultrasound frequency (Figure 3a,b). On the contrary, all the quadratic coefficients and the interaction coefficient X_2_X_3_ (ultrasound frequency and extraction duration) were negative. Thus, it clearly appeared that RA extraction according to these parameters passed by a maximum (Figure 3c). Here, prolonged extraction duration associated with high ultrasound frequency resulted in a decrease in RA content. This observed decrease could be due to partial degradation of RA. High ultrasound frequency is known to be potentially destructive and to induce oxidation of natural products that could lead to the loss of the biological activities of these compounds [25,26,27]. According to the adjusted second order polynomial equation, optimal conditions were 45 min extraction in an ultrasound bath operating at 36.8 kHz and using 99.8% (*v*/*v*) aqueous ethanol as extraction solvent. These conditions were adjusted to the material and highest RA content was therefore obtained following 45 minutes extraction in an ultrasound bath operating at 30 kHz and using pure ethanol as extraction solvent. Under these conditions, an RA content of 110.8 mg/g DW was measured from lyophilized *P. scutellarioides* leaves. The relative RA purity was estimated according to the method described by Falé et al. [11] for *P. barbatus* leaves decoction. Under our optimal extraction conditions, RA was estimated to represent around 70% of the total absorption of the wavelength using PDA detection. This was further confirmed by DEDL detection (data not shown). Our optimal conditions are quite similar to those described by Caleja et al. [34] for the USAE of RA from *R. officinalis* leaves, but comparatively our results showed a higher extraction yield with *P. scutellarioides* leaves as starting material. In the literature, RA extraction yields from *Lamiaceae* leaves using USAE greatly varied [40,41], ranging from 0.01 mg/g DW in *H. cuspidatus* [31] to 86.3 mg/g DW in *M. officinalis* [34], whereas a higher yield of 12.6 mg/g DW was obtained using *Marantha depressa* (*Maranthaceae*) leaves as starting material [42]. Comparatively, our results are in the high range of these values.

### 2.3. Validation of the Extraction Method

Quantification of RA was achieved by HPLC analysis (Figure S2) and RA was identified by comparison with an authentic standard. A good separation resolution must be one of the primary goal for quantitative HPLC analysis. For this purpose, a separation resolution (R_S_) of at least 1.5 should be obtained, with the exception of samples containing a high number of component, which is the case of raw plant extracts, or compounds more difficult to separate such as isomers [43]. Here, the presence of *cis*-RA, an isomer of *trans*-RA, resulted in a separation resolution of ca 1.3 (1.298). Considering that we here work with a raw plant extract and the nature of the ‘interfering’ peak, this resolution (higher than 1.0) is therefore acceptable according to international standards [43]. To ensure the accuracy and precision of the method used to quantify RA, the HPLC method was validated. The validation parameters are presented in Table 5.

The six-point calibration curve of the peak areas (y) against the injected quantities of RA at 320 nm was linear over the wide range analyzed (50–1000 µg/ml) with R^2^ > 0.999 and the slope of the standard covering the analytical range varied at most 1% relative standard deviation (RSD) over a four-week period. The LOD (S/N = 3) and the LOQ (S/N = 10) were as low as 1.8 ng and 5.3 ng. Then, the instrumental precision determination was obtained through five injections of the same sample. The method precision and stability were confirmed by the low RSD values of 0.48% and 0.94% obtained for interday and intraday precisions respectively (Table 5). The repeatabilty of the method was evidenced with a RSD value as low as 3.5%, as well as its accuracy, determined by four levels of h standard addition (from 5 to 50 µg/mL) showing a good recovery with a mean for the RSD values of 2.8% (Table 5).

### 2.4. Biological Activities of the Extracts

**Figure 4:**
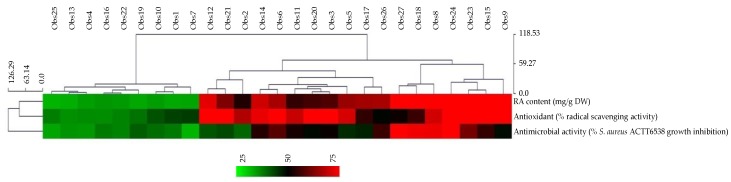
Hierarchical clustering analysis of RA contents and antioxidant (DPPH radical scavenging activity) and antimicrobial (growth inhibition of *S. aureus* ACTT6538) of the 27 extracts obtained following USAE of *P. scutellarioides* leaves.

The next steps were the evaluation of the antioxidant and antimicrobial activities of the 27 extracts obtained using the full factorial design in order to confirm that the extraction did not alter the biological properties of RA and to establish correlations between these biological activities and the RA content. For this purpose, antioxidant activity was determined by the radical scavenging activity using the commonly used DPPH in vitro assay [44], whereas antimicrobial activity was determined by monitoring the growth inhibition of pathogenic strain of *Staphylococcus aureus* strain ACTT6538 using microplate method described by El Abdellaoui et al. [45]. The results are presented as a heatmap representation and classified using a hierarchical clustering analysis (HCA) using Euclidian distance (Figure 4). The individual values are presented in Table S1.

Surface response plots showing the impact of the three independent parameters (ethanol concentration, ultrasound frequency, and extraction duration) are presented in Figure S3 for the radical scavenging activity and Figure S4 for the antimicrobial activity. These 3D plots depicted a very similar aspects to those obtained for RA extraction (Figure 3). Strong significant correlations were observed between RA content and antioxidant activity (Pearson coefficient correlation (PCC) = 0.836, *p* = 0.004 **) as well as between RA content and antimicrobial activity (PCC = 0.822, *p* = 0.017 *) were calculated confirming that the applied USAE conditions maintained RA into a bioactive form and preserved the associated biological activities. In particular the extract obtained using optimal conditions, containing the highest RA content, here offered the best radical scavenging activity (89.7% inhibition of radical formation) and antimicrobial action toward *S. areus* (79.8% growth inhibtion).

### 2.5. Comparison with Conventional Extraction Protocol and Other Biotechnological Sources

The last step of this work was to compare the optimal USAE of RA from *P. scutellarioides* leaves with a conventional heat reflux extraction method. For this purpose the same ethanol concentration (pure ethanol), temparature (45°C), and solid-to-liquid ratio (25:1) conditions were used, but using three different extraction duration of 45 min (i.e., the same extraction time used for the optimal USAE of RA), 90 min (i.e., 2-times the duration used for the optimal USAE of RA), and 180 min (i.e., 4-times the duration used for the optimal USAE of RA). The RA contents obtained are presented in Table 6.

The results of this comparison clearly indicated that USAE provided better RA extraction yield and in a shorter time compared with the conventional heat reflux method. Using the conventional heat reflux method, the RA content increased with extraction duration, but even after 180 minutes, the extraction yield is still lower than for USAE with a 3-times reduced extraction time. Note that for the same extraction duration the RA content obtained using the conventional heat reflux method is around half of this obtained using USAE. These results clearly evidenced that the use of USAE to extract RA from *P. scutellarioides* leaves is of great interest, in particular within the context of green chemistry, in term of reducing energy consumption by using this innovative technology. Here, USAE increased RA extraction yield and therefore lowered extraction costs (due to the reduction of treatment time and solvent consumption) certainly as a consequence of solvent heating by directly interacting with the water molecules present in the plant tissue resulting in a more efficient rupture of these tissue and release of RA into the solvent as it was hypothetized for other plants [25,26,27].

Many studies reported on RA production from *P. scutellarioides* using different biotechnological systems to improve its production from *in planta* using in vitro technologies, but little attention has been paid to the optimization of its extraction directly from the leaves of this ornamental plant. Here, using our optimized and validated USAE method, we report on RA yield of 11.08% DW from the leaves as starting material which is in the range of RA production obtained using biotechnological systems. Indeed, several authors reported on RA contents ranging from 2.85% (DW) following permeabilization using DMSO [30] to 19.00% (DW) after 4% sucrose feeding [29] using cell suspensions, or of 7.60% (DW) using hairy roots subjected to methyl jasmonate elicitation [28]. Considering *P. scutellarioides* as an ornamental plant that could easily be multiplied by cuttings, and its leaves are a cheap and renewable material, the present USAE method of RA, using *P. scutellarioides* leaves as raw starting material, is therefore of great interest for feasible industrial applications.

## 3. Materials and Methods

### 3.1. Chemicals and Reagents

Analytical grade extraction solvents were used in the present study and were obtained from Fisher Scientific (Illkirch, France). RA standard was purchased from Sigma-Aldrich (Saint-Quentin Fallavier, France). All chemicals for the purpose of antioxidant and antimicrobial activities were from Sigma-Aldrich (Saint-Quentin Fallavier, France).

### 3.2. Plant Materials

*Plectranthus scutellarioides* (L.) R.Br., seeds (commercial cultivar “Arc-en-Ciel Amélioré”) were obtained from Vilmorin-Mikado (La Ménitré, France). The plantlets resulting from seedlings were cultivated grew in pots (30 cm diameter and 30 cm depth), filled with a commercial garden soil (N: 250 g/m^3^, P2O5: 120 g/m^3^, K2O: 80 g/m^3^, 37% of dry matter, 65% of organic matter, pH: 6.2, conductivity: 49 mS/cm, water retention capacity: 70% vol.) in a phytotronic room at 25°C under a 16-h photoperiod (30 µmol/m^2^/s total amount of photosynthetically active radiation) and the relative humidity (RH) was approximately 30%. Plants were irrigated using overhead mist irrigation once a day and one complete watering per week. Three-month-old leaves (just before flowering in order to limit possible remobilization of RA from leaves to seeds) were collected, stored at −80°C and lyophilized prior to extraction.

### 3.3. Ultrasound-Assisted Extraction (USAE) Optimization

USAE was performed using an ultrasonic bath USC1200TH (Prolabo, Sion, Switzerland) with an inner dimension of 300 × 240 × 200 mm, an electrical power of 400W (i.e., acoustic power of 1W/cm^2^), maximal heating power of 400W, variable frequencies, equipped with a digital timer, a frequency and a temperature controller. The sample was placed in a 100-ml quartz tube topped by a vapor condenser and was suspended in 20 ml aqueous ethanol. For this purpose, various solvent to material (S/M) ratios were evaluated (10:1, 25:1, and 50:1 ml/g DW) and various aqueous ethanol concentrations (0, 25, 50, 75, and 100 % (*v*/*v*)) were evaluated. Different ultrasound frequencies (0, 15, 30, and 45 kHz), extraction temperatures (25, 35, 45, 55, 65, and 75°C), and extraction durations (0, 15, 30, 45, 60, and 75 min) were also tested. The extract was then centrifuged for 15 min at 3,000 rpm and the supernatant was filtered (0.45 μm) before HPLC analysis.

### 3.4. Conventional Solid/Liquid Extraction

The conventional heat reflux extraction consisted in S/M ratio of 25:1 ml/g DW using 20 ml of pure ethanol (100%) as the extraction solvent, in a water bath with extraction temperature set at 45°C during 45, 90, and 180 min. After these extraction times, the extract was centrifuged at 3,000 rpm for 15 min, and the resulting supernatant filtered (0.45 μm) prior HPLC analysis.

### 3.5. High-Performance Liquid Chromatography (HPLC) Analysis

Separation was performed using Varian (Les Ulis, France) high-performance liquid chromatography system equipped with Varian Prostar 230 pump Metachem Degasit, Varian Prostar 410 autosampler and Varian Prostar 335 Photodiode Array Detector (PAD) and driven by Galaxie version 1.9.3.2 software. Hypersil PEP 300 C18 (Thermo Fisher Scientific, Illkirch, France), 250 × 4.6 mm, 5 µm particle size equipped with a guard column Alltech (Thermo Fisher Scientific, Illkirch, France), 10 × 4.1 mm was utilized at 35°C was used for the separation. The mobile phase was composed of HPLC grade solvents: A is a mixture of HCOOH/H_2_O at pH 2.1 and B is CH_3_OH. Throughout one-hour run, mobile phase composition varied with a nonlinear gradient 8% B (0 min), 12% B (11 min), 30% B (17 min), 33% B (28 min), 100% B (30–35 min), 8% B (36 min) at a flow rate of 1 ml/min. A 10-min re-equilibration time was used among individual runs. The detection of compounds was set at 320 nm. Quantification was done based on assessment of retention time and UV spectrum of a reference standard purchased from Sigma-Aldrich (Saint-Quentin Fallavier, France). The samples examination was done in triplicates.

### 3.6. Experimental Design

Factorial experiment design and response surface plots were used to identify the optimal RA extraction conditions using XLSTAT2018 software (Addinsoft, Paris, France). Variables were coded at three levels −1, 0, and +1. The three independent variables and their values were selected following preliminary experiments: X_1_ ethanol concentration (50, 75, and 100% *v*/*v*), X_2_ ultrasound frequency (15, 30, and 45 kHz) and X_3_ extraction duration (30, 45, and 60 min). Twenty-seven observations of different combinations were prepared by taking values of selected variables at different levels as shown in Table 2. Note that all independent observations were carried out in triplicate. The equation calculation as well as statistical analysis were performed as described previously [24,46].

### 3.7. Method Validation

The method was then validated following the recommendations of the association of analytical communities (AOAC) to ensure the precision and accuracy of the method used to quantify RA.

Five-point calibration curves were made by means of diluted solutions of RA authentic commercial standard (Sigma Aldrich, Saint-Quentin Fallavier, France). Each sample was injected three times and arithmetic means was calculated to generate linear regression equations plotting was done by the peak areas (y) against the injected quantities (x) of RA standard compound. Coefficients of determination (R^2^) was used for linearity verification.

The signal-to-noise ratios (S:N) of 3:1 and 10:1, were used to respectively determine the limits of detection (LOD) and of quantification (LOQ).

Accuracy, method precision, stability, and repeatability were determined as described in Corbin et al. [26].

### 3.8. Antioxidant DPPH assay

The antioxidant activity was evaluated using the protocol described in [21,47].

### 3.9. Antibacterial Activity

The antibacterial activity was tested using the microplate protocol as described in [23,45] on *Staphylococcus aureus* ACTT6538.

### 3.10. Statistical Analysis

Each experiment was carried out at least in triplicate and XL-stat_2018 (Addinsoft, Paris, France) was used for all statistical analyses. All statistical tests were considered significant at *p* < 0.05.

## 4. Conclusions

Rosmarinic acid (RA) has already been demonstrated to be a promising compound for cosmetic applications. Nevertheless, its extraction was far from efficient. To date, many studies dealing with RA production from *P. scutellarioides* have focused on biotechnological approaches to improve its production from in planta using in vitro technologies, but little attention has been paid to the optimization of its extraction from the leaves of this ornamental plant. However, this plant is known as easy to propagate by cuttings and high renewable biomass production of leaves can be obtained through basic horticultural approaches. The development of green extraction of RA from this raw material in potential cosmetic applications is promising. Here we report on the development and validation of an efficient extraction procedure of the antioxidant and antimicrobial *trans*-rosmarinic acid from *P. scutellarioides* leaves. The present study describes an efficient and validated USAE method for RA extraction and quantification from *P. scutellarioides* leaves. A maximum RA of 11.08 % of *P. scutellarioides* leaves DW was obtained using pure ethanol a green extraction solvent in an ultrasonic bath operating at a 30 kHz frequency during 45 min. This extraction method was validated in terms of precision, repeatability, stability, and accuracy. The proposed method was proved as more efficient and less time-consuming than the conventional heat reflux method. Comparison with RA yields obtained from different *P. scutellarioides* systems confirmed that the leaves of this ornamental plant can be used as raw starting material for an efficient extraction of RA. Thus, the present method is of particular interest within the context of green chemistry in terms of reducing energy consumption and the use of green solvent. We anticipate that it could allow fast and easy extraction of RA for future cosmetic applications.

## Figures and Tables

**Table 1:** Identities, code unit, coded levels and actual experimental values of the three independent variables.

Independent variable	Code unit	Coded variable levels		
		−1	0	+1
Ethanol concentration (% *v*/*v*) ^1^	X_1_	50	75	100
US frequency (kHz)	X_2_	15	30	45
Extraction duration (min)	X_3_	15	30	45

^1^ % of ethanol concentration in mixture with HPLC grade ultrapure water.

**Table 2:** Results of experimental design.

Run ID	Run order	X_1_	X_2_	X_3_	RA (mg/g DW)
Obs1	17	−1	−1	−1	16.9 ± 2.6
Obs2	24	0	−1	−1	53.2 ± 0.5
Obs3	26	+1	−1	−1	57.8 ± 6.0
Obs4	21	−1	0	−1	18.7 ± 3.8
Obs5	22	0	0	−1	65.2 ± 5.0
Obs6	6	+1	0	−1	66.5 ± 2.4
Obs7	10	−1	+1	−1	17.6 ± 4.3
Obs8	27	0	+1	−1	78.2 ± 1.1
Obs9	7	+1	+1	−1	102.2 ± 1.15
Obs10	18	−1	−1	0	19.2 ± 2.2
Obs11	12	0	−1	0	55.1 ± 2.5
Obs12	8	+1	−1	0	72.6 ± 0.6
Obs13	25	−1	0	0	15.6 ± 0.1
Obs14	1	0	0	0	70.4 ± 7.4
Obs15	16	+1	0	0	91.6 ± 3.1
Obs16	23	−1	+1	0	20.3 ± 2.5
Obs17	11	0	+1	0	66.3 ± 2.6
Obs18	14	+1	+1	0	90.2 ± 1.3
Obs19	15	−1	−1	+1	16.9 ± 4.3
Obs20	3	0	−1	+1	56.7 ± 1.9
Obs21	13	+1	−1	+1	62.6 ± 1.6
Obs22	9	−1	0	+1	19.5 ± 0.5
Obs23	5	0	0	+1	91.1 ± 4.4
Obs24	19	+1	0	+1	110.8 ± 4.5 *
Obs25	4	−1	+1	+1	14.4 ± 1.1
Obs26	20	0	+1	+1	66.7 ± 3.0
Obs27	2	+1	+1	+1	75.9 ± 2.6

Values are the mean ± RSD of three independent replicates except for *, which correspond to the highest RA content here determined by six independent experiments to confirm this value.

**Table 3:** Statistical analysis of the regression coefficients.

Source	Value	SD	*t*	*P* > |*t*|
Constant	73.22	5.279	13.871	< 0.0001 ***
X_1_	31.74	2.444	12.988	< 0.0001 ***
X_2_	6.70	2.444	2.743	0.014 *
X_3_	2.15	2.444	0.879	0.392 ^ns^
X_1_^2^	−17.60	4.232	−4.159	0.001 **
X_2_^2^	−8.68	4.232	−2.052	0.046 *
X_3_^2^	−0.65	4.232	−0.153	0.880 ^ns^
X_1_X_2_	6.34	2.993	2.118	0.049 *
X_1_X_3_	2.11	2.993	0.707	0.489 ^ns^
X_2_X_3_	−4.11	2.993	−1.373	0.188 ^ns^

SD standard error; *** significant *p* < 0.001; ** significant *p* < 0.01; * significant *p* < 0.05; ns not significant.

**Table 4:** ANOVA of the predicted model for USAE of RA from freeze-dried *P. scutellarioides* leaves.

Source	Sum of square	df	Mean of square	*F*-value	*p*-value
Model	22074.5	9	2452.7	22.72	< 0.0001
Lack of fit	1592.8	17	93.7	0.87	0.266
Residual	1827.1	17	107.5	-	-
Pure Error	234.3	0	-	-	-
Cor. Error	23901.6	26	-	-	-
R^2^	0.924				
R^2^ adj	0.883				
CV %	0.79				

df: degree of freedom; Cor. Error: corrected error; R^2^: determination coefficient; R^2^ adj: adjusted R^2^; CV variation coefficient value.

**Table 5:** Validation parameters of the HPLC method.

Equation	R^2^	LOD (ng)	LOQ (ng)	Precision (%RSD)		Repeatability (%RSD)	Recovery (%RSD)
				Intraday	Interday		
y = 4.872x − 0.123	0.9998	1.8	5.3	0.48	0.94	3.5	2.8

**Table 6:** Comparison between conventional heat reflux method and ultrasound-assisted extraction (USAE) of RA from *P. scutellarioides* leaves.

RA content as a function of extraction method and time	Conventional Heat Reflux Extraction			USAE
Extraction duration	45 min	90 min	180 min	45 min
RA content (mg/g DW)	55.6 ± 3.1^d^	79.7 ± 1.5^c^	95.6 ± 2.2^b^	110.8 ± 4.5^a^

Values are the mean ± RSD of three independent replicates and different letters (a, b, c, d) indicate significant differences between conditions (*p* < 0.05).

## Supplementary Materials

The following are available online at https://www.mdpi.com/2223-7747/8/3/50/s1, Figure S1 Biplot representation of the linear relation between predicted vs. measured RA contents in the 27 sample extracts. Light blue contours represented *p* = 0.05; Figure S2 Representative chromatogram of a complete HPLC analysis of an extract of *P. scutellarioides* leaves obtained following USAE showing the presence of RA as main compound. *t*-RA: trans-RA (rosmarinic acid); IS: internal standard (4-hydroxychalcone); Figure S3 Predicted surface response plots of the antioxidant activity (% of DPPH radical scavenging activity) as a function of (a) ultrasound frequency and ethanol concentration, (b) extraction duration and ultrasound frequency, and (c) extraction duration and ethanol concentration; Figure S4 Predicted surface response plots of the antimicrobial activity (% of *Staphylococcus aureus* ACTT6538 growth inhibition) as a function of (a) ultrasound frequency and ethanol concentration, (b) extraction duration and ultrasound frequency, and (c) extraction duration and ethanol concentration; Table S1 Individual antioxidant and antimicrobial activities vs. RA contents in the 27 US extract samples.
