# Supplementary material for: Green Ultrasound Assisted Extraction of trans Rosmarinic Acid from Plectranthus scutellarioides (L.) R.Br. Leaves
Source: Plants (Basel). 2019 Feb 27;8(3):50. doi: 10.3390/plants8030050 (PMC6473734; doi:10.3390/plants8030050)
Supplement: Supplementary file 1 [file plants-08-00050-s001.zip › plants-427831-SI.pdf]

Plants 2019, 8, 50; doi:10.3390/plants8030050

Article

Green Ultrasound Assisted Extraction of *trans* Rosmarinic Acid from *Plectranthus scutellarioides* (L.) R.Br. Leaves

Duangjai Tungmunnithum[1,2,3,*], Laurine Garros[1,2,4], Samantha Drouet[1,2], Sullivan Renouard[5], Eric Lainé[1,2] and Christophe Hano [1,2,*]

[1] Laboratoire de Biologie des Ligneux et des Grandes Cultures, INRA USC1328 Orleans University, 45067 Orléans Cedex 2, France ; laurine.garros@etu.univ-orleans.fr (L.G.); samantha.drouet@univ-orleans.fr (S.D.); eric.laine@univ-orleans.fr (E.L.)

[2] Bioactifs et Cosmetiques, CNRS GDR 3711 Orleans, 45067 Orléans Cedex 2, France

[3] Department of Pharmaceutical Botany, Faculty of Pharmacy, Mahidol University, Bangkok 10400, Thailand

[4] Institut de Chimie Organique et Analytique, CNRS UMR731, Orleans University, 45067 Orléans Cedex 2, France

[5] Institut de Chimie et de Biologie des Membranes et des Nano-objets, CNRS UMR 5248, Bordeaux University, 33600 Pessac, France; sullivan.renouard@u-bordeaux.fr

* Correspondence to: duangjai.tun@mahidol.ac.th (D.T.); hano@univ-orleans.fr (C.H.); Tel.: +662 6448677-91 (D.T.); Tel.: +33-237-309-753 (C.H.)

Supplementary Materials:

Figure S1 Biplot representation of the linear relation between predicted *vs* measured RA contents in the 27 sample extracts. Light blue contours represented $p = 0.05$.

Figure S2 Representative chromatogram of a complete HPLC analysis of an extract of *P. scutellarioides* leaves obtained following USAE showing the presence of RA as main compound. *t*-RA: trans-RA (rosmarinic acid); IS: internal standard (4-hydroxychalcone).

Figure S3 Predicted surface response plots of the antioxidant activity (% of DPPH radical scavenging activity) as a function of (a) ultrasound frequency and ethanol concentration, (b) extraction duration and ultrasound frequency, and (c) extraction duration and ethanol concentration.

Figure S4 Predicted surface response plots of the antimicrobial activity (% of *Staphylococcus aureus* ACTT6538 growth inhibition) as a function of (a) ultrasound frequency and ethanol concentration, (b) extraction duration and ultrasound frequency, and (c) extraction duration and ethanol concentration.

Table S1 Individual antioxidant and antimicrobial activities vs RA contents in the 27 US extract samples.

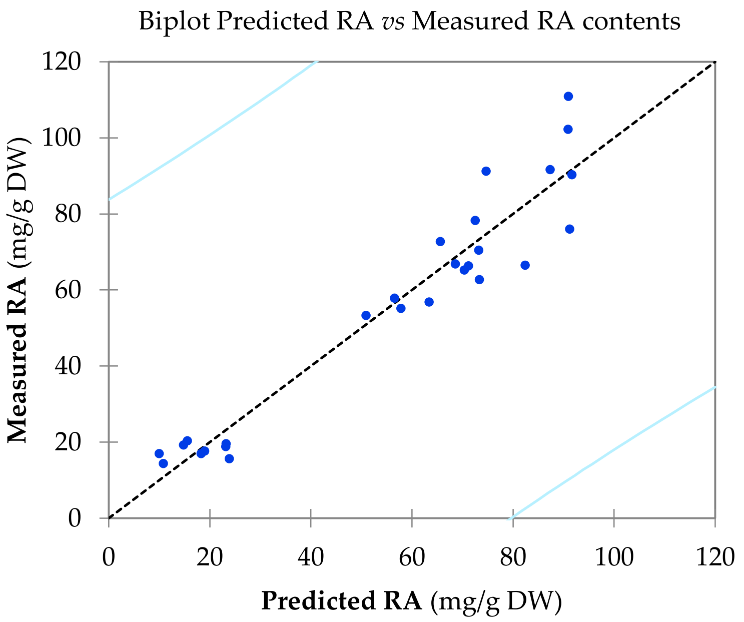

Figure S1 Biplot representation of the linear relation between predicted *vs* measured RA contents in the 27 sample extracts. Light blue contours represented $p = 0.05$.

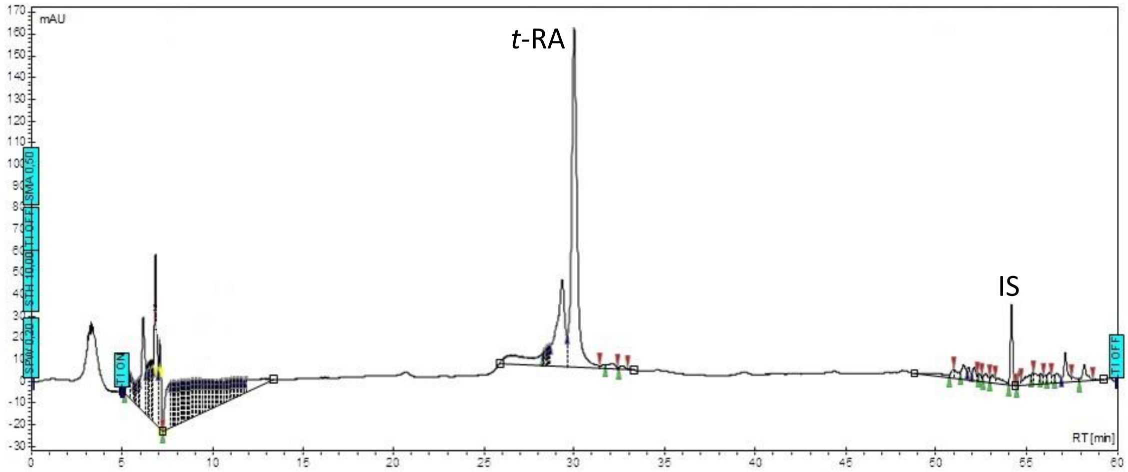

Figure S2 Representative chromatogram of a complete HPLC analysis of an extract of *P. scutellarioides* leaves obtained following USAE showing the presence of RA as main compound. *t*-RA: trans-RA (rosmarinic acid); IS: internal standard (4-hydroxychalcone).

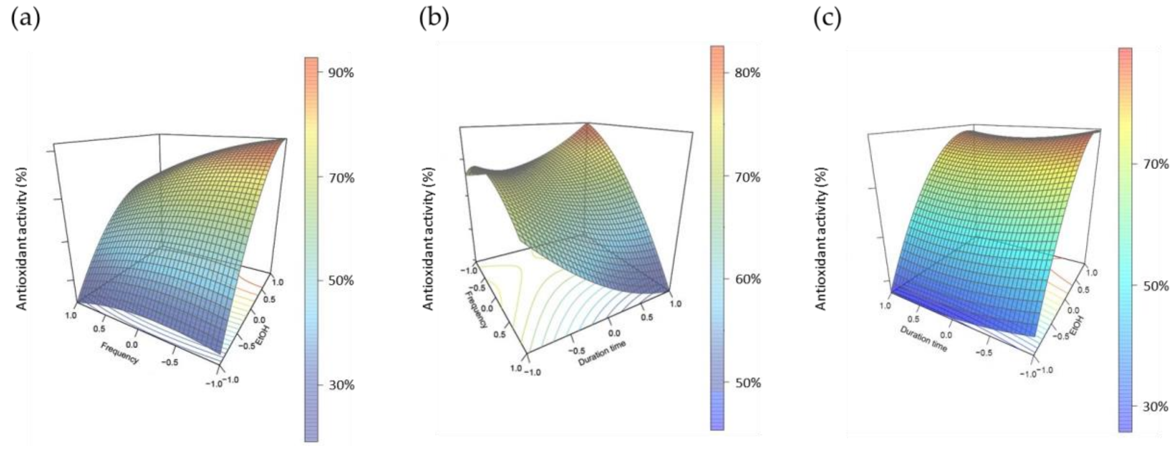

Figure S3 Predicted surface response plots of the antioxidant activity (% of DPPH radical scavenging activity) as a function of (a) ultrasound frequency and ethanol concentration, (b) extraction duration and ultrasound frequency, and (c) extraction duration and ethanol concentration.

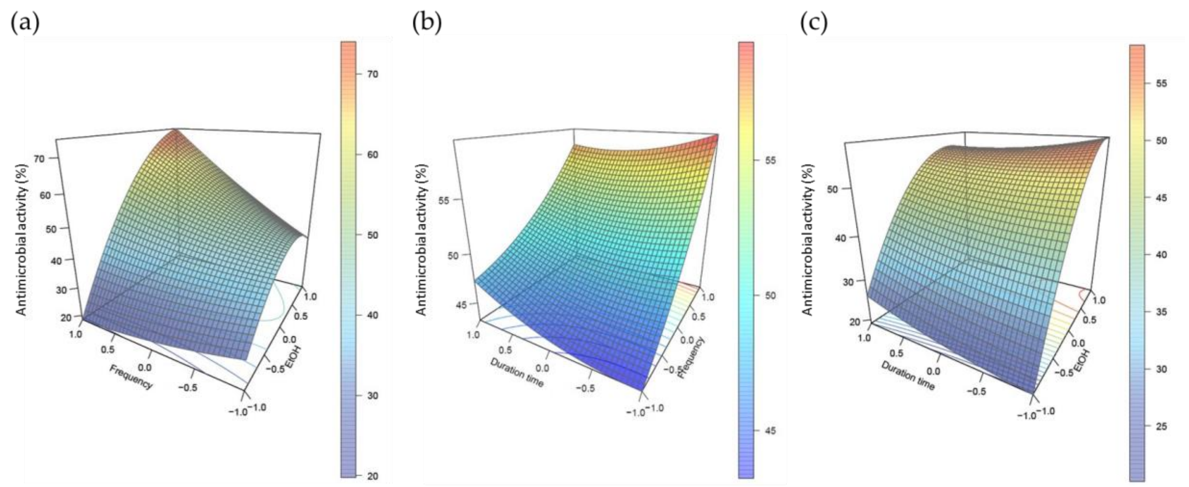

Figure S4 Predicted surface response plots of the antimicrobial activity (% of *Staphylococcus aureus* ACTT6538 growth inhibition) as a function of (a) ultrasound frequency and ethanol concentration, (b) extraction duration and ultrasound frequency, and (c) extraction duration and ethanol concentration.

Table S1 Individual antioxidant and antimicrobial activities vs RA contents in the 27 US extract samples.

Run ID[1]	Antioxidant activity[2]	Antimicrobial activity[3]	RA content (mg/g DW)
Obs1	37,31	26,09	16,88
Obs2	67,41	30,00	53,21
Obs3	79,97	51,20	57,76
Obs4	22,66	20,33	18,74
Obs5	71,26	41,52	65,15
Obs6	79,40	58,80	66,45
Obs7	38,09	14,46	17,56
Obs8	70,57	73,59	78,18
Obs9	79,03	75,11	102,18
Obs10	34,09	28,48	19,15
Obs11	69,65	52,28	55,11
Obs12	87,31	33,15	72,64
Obs13	21,71	20,76	15,60
Obs14	73,17	53,70	70,42
Obs15	85,59	55,22	91,59
Obs16	22,00	25,76	20,26
Obs17	54,98	43,37	66,28
Obs18	55,60	73,91	90,21
Obs19	27,50	31,74	16,88
Obs20	77,20	48,15	56,74
Obs21	89,77	35,54	62,61
Obs22	24,09	24,24	19,54
Obs23	80,69	61,74	91,15
Obs24	87,04	79,78	110,84
Obs25	25,25	17,61	14,34
Obs26	48,86	55,87	66,74
Obs27	51,44	47,39	75,91

Values are means of 3 independent replicates; colors represent the relative activities or contents, from blue (for relative low activities or contents) to red (for relative high activities or contents); [1] extraction conditions are described in Table 2; [2] % of DPPH radical scavenging activity; [3] % of *Staphylococcus aureus* ACTT6538 growth inhibition.
